# SGLT2 Inhibitors in Glomerulonephritis: Beyond Nephroprotection?

**DOI:** 10.3390/jcm14103533

**Published:** 2025-05-18

**Authors:** Lucia Del Vecchio, Silvia Peiti, Giulio Pucci Bella, Francesco Locatelli

**Affiliations:** 1 Department of Nephrology and Dialysis, ASST Lariana, 22100 Como, Italy; silvia.peiti@asst-lariana.it (S.P.); giulio.puccibella@asst-lariana.it (G.P.B.); 2 Department of Nephrology and Dialysis, (Past Director), ASST Lecco, 23900 Lecco, Italy; francesco.locatelli2210@outlook.it

**Keywords:** SGLT2 inhibitors, glomerulonephritis, proteinuria, chronic kidney disease, immunosuppression, lupus nephritis, IgA nephropathy

## Abstract

Sodium-glucose cotransporter 2 (SGLT2) inhibitors, initially developed for glycaemic control in type 2 diabetes, have demonstrated substantial renal and cardiovascular protective effects across various chronic kidney diseases (CKD), including glomerulonephritis. Beyond their established haemodynamic and metabolic benefits, recent evidence points to additional mechanisms of action potentially relevant to immune-mediated kidney diseases, such as the modulation of inflammation, immunometabolism, and oxidative stress. Randomised clinical trials (DAPA-CKD and EMPA-KIDNEY) and real-world observational studies consistently show that SGLT2 inhibitors reduce proteinuria and slow estimated glomerular filtration rate (eGFR) decline in patients with glomerulonephritis, including IgA nephropathy and focal segmental glomerulosclerosis. These benefits may extend to patients with stable immunosuppression. Further data are needed in this subgroup. Importantly, SGLT2 inhibitors display a favourable safety profile, even among those with immunosuppressed status. Again, further evidence is awaited in this respect. Despite these promising findings, unanswered questions remain regarding their efficacy in nephrotic syndrome, early-stage disease, and in comparison or combination with other supportive therapies. Overall, the evolving evidence supports the inclusion of SGLT2 inhibitors as a key component of supportive therapy in glomerulonephritis, with potential benefits extending beyond proteinuria reduction.

## 1. Introduction

An important goal in nephrology is the development of treatments that can prevent progression to renal replacement therapy in patients with progressive chronic kidney disease (CKD). Unfortunately, we are still far from achieving this goal. Glomerulonephritis remains one of the leading causes of end-stage kidney failure (ESKF), even though much attention has been paid to the development of innovative treatment strategies. These have two different, often overlapping, aims. The first is to block the immunological mechanism responsible for glomerulonephritis. The second is the so-called “supportive therapy”.

For many years, supportive therapy was based solely on the use of renin–angiotensin system (RAS) blockers. These drugs reduce the progression of kidney disease mainly by a haemodynamic mechanism, reducing glomerular filtration pressure (in addition to reducing systemic pressure) by vasodilation of the efferent arteriole. Their efficacy is proportional to the degree of baseline proteinuria. Although RAS inhibitors are very effective, there is a significant residual risk of progression of CKD to the point of requiring renal replacement therapy, particularly in non-proteinuric patients. Notably, this is not a typical feature of patients with glomerulonephritis.

Sodium-glucose transport 2 (SGLT2) inhibitors were initially indicated to improve glycaemic control in type 2 diabetes. They were subsequently approved for cardiac and renal protection independently of diabetes status. This is an adjunct and not a substitute therapeutic intervention to RAS inhibitors, adding further efficacy in terms of reducing proteinuria and reducing the risk of progression to ESKF or a cardiovascular event. The reduction in albuminuria observed with SGLT2 inhibitors makes these agents particularly attractive in the setting of glomerulonephritis, considering that they are usually prescribed in addition to RAS blockade.

## 2. Possible Additional Advantages of SGLT2 Inhibitors in Glomerulonephritis

It has already been clearly established that SGLT2 inhibitors exert nephroprotective effects beyond glucose regulation and the reduction in intraglomerular pressure through tubular–glomerular feedback. SGLT2 inhibition also leads to an initial decline in eGFR in non-diabetic patients, which stabilises over time. However, patients without diabetes just physiologically filter glucose; the increased glucose delivery to the distal tubule is easily compensated by increased reabsorption through SGLT1 receptors [1]. Furthermore, the observed decrease in intraglomerular pressure due to vasoconstriction of the afferent arteriole appears mild at best in the absence of diabetes [2,3,4].

**Figure 1:**
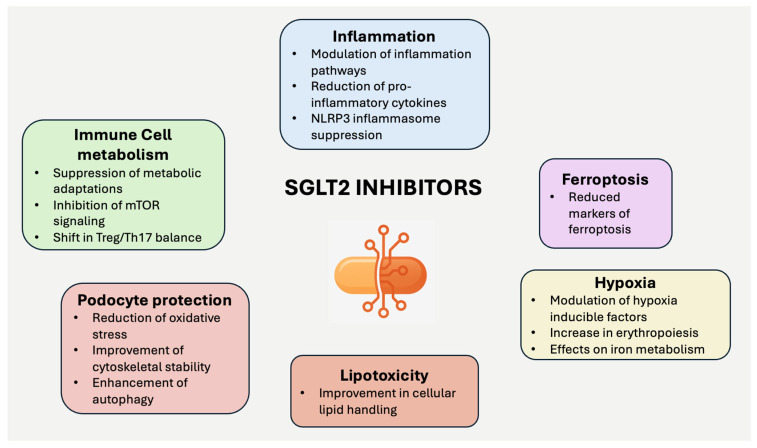
Possible beneficial effects of SGLT2 inhibitors in patients with glomerulonephritis.

Among the beneficial effects of SGLT2 inhibitors, many of them may be of particular interest in the setting of primary and secondary glomerulonephritis (Figure 1).

Inflammation plays a key role in glomerulonephritis, and SGLT2 inhibitors have been shown to modulate inflammatory pathways [5]. The rationale for this physiological process is that cytokines play a role in metabolic adaptations to changes in food intake and exercise [6].

Conversely, the inflammatory response requires increased energy substrates [7]. Several lines of experimental evidence show that these agents reduce circulating levels of pro-inflammatory cytokines such as IL-6, TNF-α, and IL-1β [8,9,10], all of which are implicated in immune-mediated kidney injury. In addition, SGLT2 inhibitors can suppress the nucleotide-binding domain-like receptor protein 3 (NLRP3) inflammasome [11,12], a key regulator of inflammation associated with ageing and heart and kidney disease progression. Notably, the inflammatory pathway is involved in the activity of some primary or secondary glomerulonephritis [13,14].

SGLT2 inhibitors also affect the metabolism of immune cells, which are crucial in the pathogenesis of autoimmune and inflammatory kidney diseases. T cells, B cells, and macrophages undergo metabolic changes during immune activation to support their proliferation and effector functions. SGLT2 inhibitors suppress these metabolic adaptations, reducing the activation and differentiation of pro-inflammatory T-cell subsets [15]. In experimental models, SGLT2 inhibitors inhibit mTOR signalling, which plays a central role in T cell activation and function [16]. In addition, these agents can shift the balance between regulatory T cells (Tregs) and pro-inflammatory Th17 cells, which may contribute to their immunomodulatory effects [17].

These preliminary data demonstrate the immunomodulatory potential of SGLT2 inhibitors.

Podocyte protection is another potential effect of SGLT2 inhibitors that is particularly interesting in some forms of glomerulonephritis. SGLT2 inhibition may reduce oxidative stress, improve cytoskeletal stability [18], and enhance autophagy [19,20], a cellular defence mechanism that maintains podocyte integrity. Experimental studies also suggest that these drugs attenuate podocyte damage in conditions of glomerular stress, including membranous nephropathy [21] and lupus nephritis [22].

SGLT2 inhibitors may affect renal oxygen consumption by modulating hypoxia-related pathways. They reduce renal cortical and medullary oxygen demand, influencing hypoxia-inducible factor (HIF) expression. These changes may have implications for the progression of kidney disease, particularly in conditions where chronic hypoxia contributes to tissue injury. In addition, SGLT2 inhibition increases erythropoiesis and modulates iron metabolism, with potential indirect benefits for maintaining renal perfusion and reducing hypoxia-induced damage. However, direct studies linking hypoxia, glomerulonephritis, and SGLT2 inhibitors are limited.

The role of ferroptosis, an iron-dependent form of cell death, is increasingly recognised in kidney diseases such as diabetic nephropathy, lupus nephritis, and IgA nephropathy (IgAN). SGLT2 inhibitors reduce markers of ferroptosis in experimental models of diabetic kidney disease [23] or the transition from acute to CKD [24]. This may also be the case in glomerulonephritis.

Lipotoxicity, characterised by the accumulation of harmful lipid metabolites, contributes to glomerular and tubulointerstitial damage. Podocytes are particularly susceptible to lipid-induced injury due to their dependence on the integrity of the slit diaphragm, which is rich in cholesterol, fatty acids, and sphingolipids. Lipid accumulation could also damage mesangial and tubular cells. Experimental studies have shown that SGLT2 inhibitors can improve lipid handling in podocytes and tubulointerstitial cells. This may attenuate lipotoxic stress in proteinuric kidney disease [25,26].

The broad metabolic and immunomodulatory effects of SGLT2 inhibitors provide a strong rationale for their potential role in these diseases. However, the precise contribution of each mechanism to the clinical benefits observed in glomerulonephritis remains to be fully elucidated.

## 3. Clinical Data from Randomised Clinical Trials and Real-World Experiences

### 3.1. Overall Effect on Glomerulonephritis

The Dapagliflozin and Prevention of Adverse Outcomes in Chronic Kidney Disease (DAPA-CKD) trial [27] and the Study of Heart and Kidney Protection with Empagliflozin (EMPA-KIDNEY) trial [28] provided strong evidence that SGLT2 inhibitors can reduce proteinuria and the risk of progression to end-stage renal disease and renal and cardiovascular death in patients with diabetic and non-diabetic CKD. Based on this evidence, the Kidney Disease Global Outcome (KDIGO) guidelines for the diagnosis and treatment of CKD recommend SGLT2 inhibitors as part of the standard of care, in addition to RAS inhibition, for a substantial percentage of patients with CKD [29].

**Figure 2:**
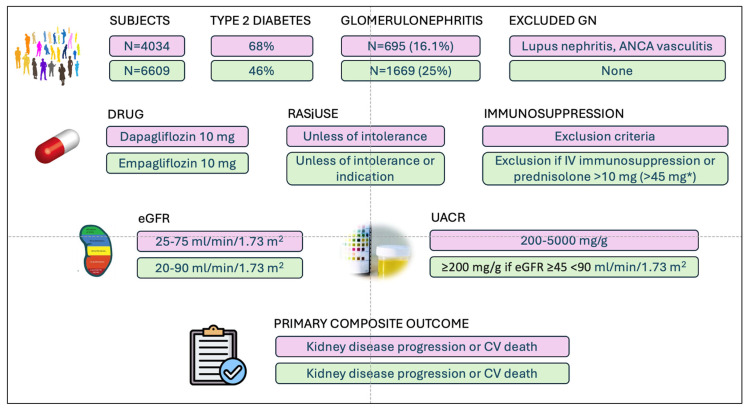
Study design of DAPA-CKD [27] and EMPA-KIDNEY [28] focusing on glomerulonephritis. CV, cardiovascular; RAS, renin angiotensin system; GN, glomerulonephritis; *, following protocol amendment.

Both trials enrolled a large number of patients with chronic glomerulonephritis, although they differed in terms of inclusion and exclusion criteria and, therefore, patient characteristics (Table 1, Figure 2).

DAPA-CKD included patients with an eGFR between 25 and 75 mL/min/1.73 m^2^, with a mean eGFR of 42.8 mL/min/1.73 m^2^ in patients with glomerular disease [30] (Figure 2). Almost two-thirds of the patients had CKD stage 3, with only 66 (9.4%) having CKD stage 1 or 2. EMPA-KIDNEY had broader eGFR inclusion criteria (Figure 2). Nevertheless, the mean eGFR (42.6 mL/min/1.73 m^2^) was similar to that in DAPA-CKD [32]. In both studies, patients with glomerulonephritis had mild proteinuria (a median urine albumin-to-creatinine ratio (UACR) of 700 mg/g (interquartile range (IQR) 306–1428) in EMPA-KIDNEY and ~975 mg/g (IQR ~533–1887) in DAPA-CKD), with little or no representation of patients with nephrotic syndrome. Some patients did not undergo renal biopsy, so the diagnosis was only clinical.

Secondary analyses of DAPA-CKD and EMPA-KIDNEY consistently showed the efficacy of SGLT2 inhibitors in patients with glomerulonephritis, with both studies confirming the efficacy of SGLT2 inhibitors in this patient population.

In DAPA-CKD, patients with glomerulonephritis randomised to dapagliflozin had a significantly lower risk of achieving the kidney-specific composite outcome compared to placebo (hazard ratio (HR) 0.43; 95% confidence interval (CI) 0.26–0.72) [30]. Similarly, dapagliflozin showed a trend towards a reduction in the risk of cardiovascular death, hospitalisation for heart failure, or all-cause mortality. However, this did not reach statistical significance because of the small number of events. This is probably because the patients with glomerulonephritis were younger and less likely to have diabetes or a history of cardiovascular disease compared to the average patient population enrolled in DAPA-CKD, suggesting a lower cardiovascular risk. The low number of events is also because the trial was stopped early due to increased efficacy. In theory, there is no reason why patients with glomerulonephritis should not have a cardiovascular benefit similar to that seen in other patient populations without diabetes.

Looking at the rate of progression as a continuous variable, the effect of dapagliflozin on the overall slope (i.e., also taking into account the initial acute drop in eGFR) in patients with glomerulonephritis was modest (−3.51 ± 0.29 mL/min/1.73 m^2^ and −3.96 ± 0.29 mL/min/1.73 m^2^ for the dapagliflozin and placebo groups, respectively). The absolute change was only 0.44 mL/min/1.73 m^2^ (95% CI 0.36 to 1.25) [33]. The effect in favour of dapagliflozin is more pronounced when considering the chronic slope alone (1.44 mL/min/1.73 m^2^ (95% CI 0.64 to 2.23); 38.4%) [31].

As for DAPA-CKD, in EMPA-KIDNEY, patients with glomerulonephritis randomised to empagliflozin had a significantly lower risk of any progression of kidney disease compared to placebo (HR 0.77, 95% CI 0.60–0.99) [34]. They also had a significantly slower mean annual rate of change in eGFR than those in the placebo group (−2.17 ± 0.16 mL/min/1.73 m^2^ and −3.60 ± 0.16 mL/min/1.73 m^2^, respectively). The absolute difference was of 1.43 mL/min/1.73 m^2^ (95% CI 0.99 to 1.87), with a relative difference of −40% (95% CI 0.52 to 0.28) [34].

Recently, long-term data from EMPA-KIDNEY confirmed the efficacy of empagliflozin in reducing the risk of the primary composite outcome of kidney disease or death from cardiovascular causes compared with placebo (HR 0.80 (0.67–0.95), including in the subgroup with glomerulonephritis [35]).

Patients with glomerular diseases treated with empagliflozin had a −1.43 slower mean chronic rate of eGFR decline than those randomised to placebo, a relative difference of −40% (−52 to −28).

Notably, the specific 95% CIs in these secondary analyses were often wide, possibly because of insufficient statistical power (both trials were stopped early for efficacy). Accordingly, the number needed to treat (NNT) in patients with glomerulonephritis was higher than expected (n = 27 for the primary outcome in EMPA-KIDNEY and n = 14 in DAPA-CKD) (Table 2).

As in diabetic or other non-diabetic kidney diseases, SGLT2 inhibitors significantly reduce proteinuria in patients with glomerulonephritis compared to placebo. However, the effect appears small (−15%; 95% CI −24 to −6% in EMPA-KIDNEY and −13.6% (−24.9 to −0.6)). Nevertheless, an exploratory analysis showed that the reduction in albuminuria was the most important determinant of the benefit observed in EMPA-KIDNEY, explaining one-fifth of the effect on chronic slope [34].

**Figure 3:**
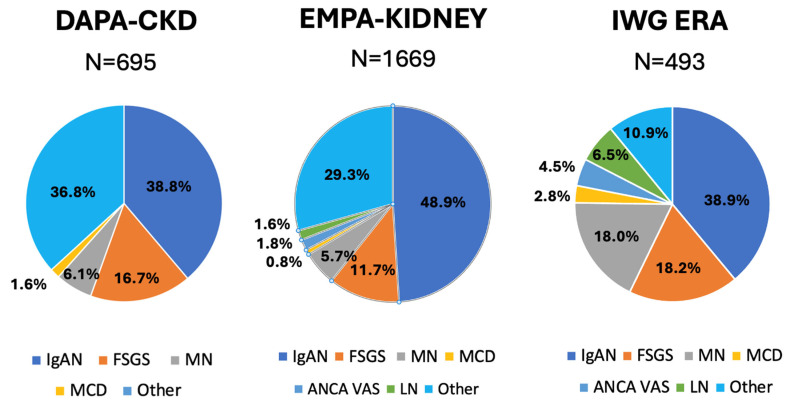
The percentage distribution of different types of glomerulonephritis in DAPA-CKD [31], EMPA-KIDNEY [34], and in observational study of the Immunonephrology Working Group of the European Renal Society [33]. IgAN, IgA Nephropathy; FSGS, focal segmental glomerulonephritis; MN, membranous nephropathy; MCD, minimal change disease; ANCA VAS, anti-neutrophil cytoplasm antibodies vasculitis; LN, lupus nephritis.

Data from everyday clinical practice are available from a collaboration of the Immunonephrology Working Group (IWG) of the European Renal Association (ERA) [33]. This retrospective international cohort included 493 patients with biopsy-proven primary and secondary glomerulonephritis (Figure 3) who were treated with SGLT2 inhibitors in addition to RAS blockade. The patients differ from those enrolled in DAPA-CKD and EMPA-KIDNEY (Table 1), with a potentially lower degree of chronicity, higher eGFR (median 56 mL/min/1.73 m^2^) and proteinuria (median 2.1 g/day, IQR 1.2–3.6, with 26% having nephrotic-range proteinuria). While DAPA-CKD and EMPA-KIDNEY excluded patients with active immunosuppression, 79 (16%) patients in this cohort were treated with maintenance immunosuppressive drugs (prednisone, n = 46; mycophenolate mofetil, n = 42; calcineurin inhibitor, n = 19; other, n = 14). The observed change in proteinuria with SGLT2 inhibitors was much greater than that reported in DAPA-CKD and EMPA-KIDNEY (−35%, −41%, −45%, and −48% at 3, 6, 9, and 12 months). The effect was consistent across histological diagnoses and irrespective of the diagnosis of diabetes. Those achieving a proteinuria decrease higher or equal to 30% also had a significant trend for a slower eGFR decline over time. A total of 153 patients (31%) had a mild degree of proteinuria reduction (<30%). The fact that this was more frequent in patients with lower serum albumin suggests that SGLT2 inhibitors may be less effective in patients with nephrotic syndrome.

One-third of the enrolled patients were obese and/or had diabetes. Interestingly, SGLT2 inhibitors had more efficacy in reducing proteinuria in those with a higher body mass index (BMI).

### 3.2. Evidence in Single Glomerulonephritis

Prespecified secondary analyses are available for IgAN and FSGS. Other primary forms of glomerulonephritis are less well represented (Figure 3). In particular, membranous nephropathy represented only 6.1% (n=) and 5.7% (n=) of the whole cohorts with glomerulonephritis enrolled in DAPA-CKD and EMPA-KIDNEY, respectively. More information on this nephropathy is available from the observational study of the IWG of ERA, which included 89 patients with membranous nephropathy (18% of the whole cohort) [30]. In these subjects, treatment with SGLT2 inhibitors was associated with a mean proteinuria decrease of –32% (95% CI –56 to 6).

Data specific to less-represented glomerulonephritis have also been reported only by the IWG of ERA, such as minimal change disease, C3 nephropathy, immune complex–mediated membranoproliferative glomerulonephritis, fibrillary glomerulonephritis, and AL amyloidosis [33]. The antiproteinuric effect was consistent in all these nephropathies, averaging between –30 and −40%. However, the CIs were very wide, possibly due to the small number of patients in each subgroup (minimal change disease, n = 14; C3 nephropathy, n = 3; immune complex-mediated membranoproliferative glomerulonephritis n = 18; fibrillary glomerulonephritis, n = 6; AL amyloidosis, n = 8).

Conversely, more data on SGLT2 inhibitor use are accumulating for lupus nephritis and ANCA vasculitis.

#### 3.2.1. IgA Nephropathy

DAPA-CKD and EMPA-KIDNEY enrolled a consistent number of patients with IgAN (270 and 817, respectively). This large sample size provides robust evidence, even when based on prespecified subgroup analyses.

In DAPA-CKD, IgAN patients were predominantly male (67.4%), with a high proportion of Asian patients (58.9%) [36]. Data on the risk of achieving the primary composite outcome (sustained ≥50% decline in eGFR, development of ESKF, eGFR <15 mL/min/1.73 m^2^, or death from renal or cardiovascular causes) were noteworthy. Only 6 (4%) of 137 patients randomised to dapagliflozin achieved the composite outcome, compared with 20 (15%) of 133 patients in the placebo group (HR 0.29; 95% CI, 0.12–0.73). Similar results were seen for the composite renal endpoint (HR 0.24; 95% CI, 0.09–0.65). The effect was consistent across subgroups of prespecified baseline eGFR and UACR categories. Treatment with dapagliflozin also slightly reduced the rate of eGFR decline compared to placebo (least mean squares eGFR slopes from baseline to end of treatment in the dapagliflozin and placebo groups of −3.5 and −4.7 mL/min/1.73 m^2^ per year, respectively; 95% CI, 0.12 to 2.51 mL/min/1.73 m^2^). The rate of eGFR decline achieved with dapagliflozin remains significantly faster than the rate of progression now considered the target for remission (i.e., below 1 mL/min/1.73 m^2^ yearly) [37]. When the initial drop in eGFR at the start of treatment of −3.4 mL/min/1.73 m^2^ is removed, the effect is more significant. The mean annualised eGFR becomes −2.2 and −4.6 mL/min/1.73 m^2^ for dapagliflozin and placebo, respectively, with a mean between-group difference of 2.4 mL/min/1.73 m^2^. The chronic slope is similar to that achieved with other treatment strategies for IgAN, such as systemic steroids, budesonide, or sparsentan [38]. Conversely, the rate of progression in the placebo group may have been faster than that typically observed in IgAN patients receiving so-called “supportive” care.

The subgroup of IgAN patients was characterised by relatively mild proteinuria (median UACR at baseline around 900 mg/g). Compared to the placebo, patients in the dapagliflozin group had a 26% greater reduction in UACR during follow-up −26% (95% CI, −37.0% to −14.0%; *p* < 0.001).

A secondary analysis specific to IgAN is not available for EMPA-KIDNEY. Data from a prespecified analysis by primary kidney disease showed that a lower number of IgAN patients randomised to empagliflozin achieved the outcome of kidney disease progression compared to those in the placebo group (51/413 vs. 67/404, respectively; HR 0.67, 95% IC 0.46–0.97) [32]. When progression was considered as a continuous variable, subjects randomised to empagliflozin had a 1.14 (0.54 to 1.75) mL/min/1.73 m^2^ lower rate of decline in the overall slope of eGFR, with patients receiving dapagliflozin losing −2.87 mL/min/1.73 m^2^ per year and those in the placebo group losing −4.01 mL/min/1.73 m^2^ per year. The chronic slope was −2.33 mL/min/1.73 m^2^ and −4.12 mL/min/1.73 m^2^ in the empagliflozin and placebo groups, respectively (difference of 1.79 mL/min/1.73 m^2^). Data on proteinuria specific to patients with IgAN were not reported.

In the real-world study of the IWG of ERA, patients with IgAN or IgA vasculitis represented 39% (139 of 493) of the total cohort [33]. At 3 months, these patients had a reduction in proteinuria of −34% (95% CI −19 to −49), similar to that seen in other cases of glomerulonephritis.

#### 3.2.2. Focal Segmental Glomerulosclerosis

Focal segmental glomerulosclerosis (FSGS) was the second most common glomerulonephritis in DAPA-CKD. This subgroup of 104 patients was the subject of a prespecified secondary analysis [39]. At baseline, patients had a mean eGFR of 41.9 mL/min/1.73 m^2^ and a median (interquartile range) UACR of 1248 (749–2211) mg/g, with patients in the placebo groups having higher values (1410 (769–2170) mg/g and 997 (736–2290) mg/g, respectively). Many subjects were obese or overweight (mean BMI 29.6 ± 6.1 kg/m^2^) and a significant proportion (19.6%) were diabetic. Consistent with the findings in the overall glomerulonephritis population, subjects randomised to dapagliflozin had a lower number of events related to the primary composite endpoint compared to those in the placebo group (4 (8.9%) and 7 (11.9%), respectively). However, the HR was not statistically significant (HR 0.62, 95% CI 0.17, 2.17), possibly because of the small number of events in both groups. Subjects in the dapagliflozin group also had a slower rate of eGFR decline (chronic slope of −1.9 (−3.0, −0.9) and −4.0 (−4.9, −3.0) mL/min/1.73 m^2^/year, respectively). Notably, in patients receiving dapagliflozin, each early 10% reduction in UACR was associated with a subsequent mean slower decline in chronic slope of −0.67 (95% CI −0.93, −0.42) mL/min/1.73 m^2^/year. However, the antiproteinuric effect in FSGS patients was mild (with a geometric mean change from baseline of −26.1% (95% CI −35.2, −15.6) and −9.9% (95% CI −19.8, 1.1) in the dapagliflozin and placebo groups, respectively). It appeared to disappear after one year of follow-up.

As with IgAN, there are no prespecified secondary analyses for FSGS in EMPA-KIDNEY. According to the published data, more subjects randomised to empagliflozin achieved the primary composite endpoint than those in the placebo group (17 (17%) and 13 (13%), respectively; HR 1.35 (95% CI 0.65–2.81)). As with DAPA-CKD, the number of events was relatively small. Similarly, the difference in eGFR decline did not reach statistical significance (−22% (95% CI −60 to 16)). However, it should be noted that the evaluation of treatment efficacy in FSGS is complex due to the heterogeneity of this type of glomerulosclerosis. Indeed, a similar inconsistency was observed for sparsentan compared to IgAN [40]. In the real-world data of the IWG, SGLT2 inhibitors were slightly more effective, achieving a −30% reduction in proteinuria (95% CI −2 to −51) [33]. However, a significant proportion (64%) had secondary forms, which may be more sensitive to agents acting on metabolic pathways [33].

#### 3.2.3. Secondary Glomerulonephritis

Recently, the possibility of prescribing SGLT2 inhibitors to patients with autoimmune diseases has gained popularity. These patients not only face an increased risk of adverse renal outcomes but also carry the burden of higher cardiovascular risk compared to subjects of similar age and sex. Experience with SGLT2 inhibitors in patients with autoimmune disease and renal involvement is still limited. Indeed, this was an exclusion criterion in DAPA-CKD, while EMPA-KIDNEY included only 58 patients with lupus nephritis and ANCA microscopic polyangiitis (Figure 1). In contrast, these two diseases were slightly better represented in the observational study of the European Renal Association Immunonephrology Working Group (ANCA-associated vasculitis n = 22 4%; lupus nephritis n = 32, 7%) (Figure 3). Although based on a small number of patients, SGLT2 inhibitors showed a relevant but not statistically significant antiproteinuric effect in both nephritides (−43% (95% CI −50 to 1) in lupus nephritis and −31% (95% CI −53 to 18) in patients with ANCA-associated vasculitis.

Some case series with lupus nephritis are reported in the literature, showing contrasting effects on proteinuria reduction [22,41]. Furthermore, preliminary data for dapagliflozin use are available from a small, single-arm, open-label, phase I/II trial of 38 Chinese patients with systemic lupus erythematosus (SLE) [42]. SLE Disease Activity Index scores and proteinuria did not improve in those with lupus nephritis. However, the prednisone dose was decreased by 30% overall. The eGFR remained stable or slightly decreased in those with a baseline eGFR of <90 mL/min/1.73 m^2^ during the 6-month follow-up period. Yen et al. [43] compared SGLT2 inhibitor users and non-users among patients with SLE associated with type 2 diabetes. They found that those treated with SGLT2 inhibitors had a lower risk of developing lupus nephritis (HR, 0.55; 95% CI, 0.40–0.77), requiring dialysis (HR, 0.29; 95% CI, 0.17–0.48), requiring kidney transplantation (HR, 0.14; 95% CI, 0.03–0.62), or experiencing heart failure (HR, 0.65; 95% CI, 0.53–0.78), or all-cause mortality (HR, 0.35; 95% CI, 0.26–0.47). Similarly, MA et al. [44] performed a population-based target trial emulation in patients with SLE and diabetes as a comorbidity. A total of 2165 patients were treated with SGLT2 inhibitors, and 2165 patients started dipeptidyl peptidase 4 (DPP4) inhibitors. The two cohorts were matched with propensity scores. Over a mean follow-up period longer than two years, the subjects treated with SGLT2 inhibitors had a significantly lower risk of developing acute kidney injury (HR 0.49, 95% CI 0.39–0.63), CKD (HR 0.61, 95% CI 0.50–0.76), ESKF (HR 0.40, 95% CI 0.20–0.80), or heart failure (HR 0.72, 95% CI 0.56–0.92). Conversely, no difference was observed in the risk for all-cause mortality, lupus nephritis, cardiovascular events, or hospitalisations.

Experience with ANCA-associated vasculitis is very limited, apart from the small cohorts enrolled in EMPA-KIDNEY and the observational study of the IWG. In this latter study [33], the 22 subjects with ANCA-associated vasculitis had a non-statistically significant decrease in proteinuria of –31% (95% CI –53 to 18).

## 4. Safety Issues Specific to Patients with Glomerulonephritis

Clinical trials have consistently demonstrated the excellent tolerance and safety profile of SGLT2 inhibitors in patients with CKD. The profile is often comparable to a placebo. However, patients in everyday clinical practice are often frailer than those enrolled in clinical trials. This could be particularly true for those of older ages or being treated with immunosuppression for a long period.

Despite the initial great concern about urinary tract infections due to glycosuria, the actual risk of severe episodes in patients without diabetes is minimal. The same holds for the risk of severe genital infections. More than that, even if SGLT2 inhibitors cause an initial fall in eGFR at the treatment start, the clinical trials showed that acute kidney injury is less frequent than in the placebo groups. Also, dehydration is rare, as the diuretic activity of SGLT2 inhibitors is mild and self-limiting. Nevertheless, this possibility should be adequately managed, especially in patients already treated with other diuretics.

Euglycemic ketoacidosis has been described during therapy with SGLT2 inhibitors; this severe adverse event could be avoided by counselling patients, especially the diabetic ones, to follow the so-called “sick day rule” and to withhold SGLT2i therapy during sickness [45].

Secondary analyses of DAPA-CKD showed that SGLT2i are safe in patients with glomerular disease [30].

IgAN patients receiving dapagliflozin experienced fewer serious adverse events compared to the placebo group, with no cases of ketoacidosis or hypoglycaemia. The number of adverse events leading to study drug discontinuation was similar between the groups [36]. Similarly, the subgroup with FSGS receiving dapagliflozin experienced fewer significant adverse events compared to placebo, with similar discontinuation rates [39].

The safety of SGLT2 inhibitors specific to other glomerular diseases has been less investigated in DAPA-CKD due to the small number of participants with any single form of glomerulonephritis.

Secondary analyses of the IgAN or FSGS population involved in EMPA-KIDNEY are still unavailable. However, the incidence of serious urinary tract infection, serious dehydration, severe hypoglycaemia, liver injury, and bone fractures were similar between the group receiving empagliflozin and placebo, irrespective of the primary kidney disease [32].

Real-world clinical data further support this evidence, showing a minimal risk of developing adverse events. In the IWG study [33], the treatment was generally well tolerated, with only a small number of patients needing to discontinue the drug due to recurrent bacterial urinary tract infections (n = 4), genital mycotic infections (n = 2), or gastrointestinal intolerance (n = 5). Notably, in this cohort, 16% of the patients received maintenance immunosuppression.

While the safety of SGT2 inhibitors in patients with glomerular disease is well established, their use in patients with active immunosuppressive therapy still requires further evaluation. Indeed, DAPA-CKD and EMPA-KIDNEY excluded patients with recent immunosuppression.

A small Egyptian study specifically examined the safety of SGLT2 inhibitors combined with immunosuppression in 17 patients with various glomerular diseases [46]. During follow-up, the authors reported two cases of symptomatic lower urinary infections successfully treated with antibiotics. In both cases, discontinuing immunosuppression or SGLT2 inhibitors was not required. No patients experienced a serious adverse event.

Some data regarding lupus nephritis come from a small Chinese study showing that dapagliflozin had an acceptable safety profile [42]. In particular, the incidence of urinary tract infections was similar to that of patients without SLE. Notably, the authors reported one case of fungal pneumonia.

An emulated clinical trial compared the safety of SGLT2 inhibitors with DPP4 inhibitors in SLE patients with type 2 diabetes [44]. Patients treated with SGLT2 inhibitors had an increased risk of genital infections. Conversely, no difference was observed for diabetic ketoacidosis, urinary tract infections, or fracture risk.

The latter point is important, as combining SGLT2 inhibitors with steroids could theoretically increase fracture risk, considering their effect on the tubular transport of calcium, phosphate, bicarbonate, and magnesium. Although this has not been confirmed in population studies [47,48], caution should be used in patients who have just received or are on active treatment with steroids. At present, no specific data are available on this point for patients with glomerulonephritis, as SGLT2 inhibitors are not used yet in combination with steroids on a large scale.

Another possible safety issue concerning SGLT2 inhibitors is polyglobulia.

It is well documented that gliflozins are associated with increased renal production of erythropoietin by reducing tubular work and oxygen requirements and possibly reducing the damage associated with hypoxic tubular cells [49]. This leads to increased haemoglobin levels in patients, regardless of whether they are anaemic or not. The effect has been documented in clinical trials [50] and real-world data [51].

In a small percentage of cases, patients develop polyglobulia, with haematocrit levels exceeding 49% in men and 48% in women [52]. A small Italian observational study evaluated the efficacy and safety of SGLT2 inhibitors in 21 patients with IgAN [53]. Alongside the well-documented increase in haemoglobin levels, three patients had new onset erythrocytosis with haematocrit levels >53%. The haematological work-up did not demonstrate a clonal disease; polycythaemia reversed following treatment discontinuation.

Whether erythrocythaemia is a true safety issue in patients not having polycythaemia vera is unknown. Available data from the literature do not suggest an increased thrombotic risk in patients treated with SGLT2 inhibitors. Accordingly, withholding the drug should be carefully balanced with treatment indications and expected benefits. Oppositely, SGLT2 inhibitors could mask anaemia and delay diagnostic evaluation in patients with lower–normal haemoglobin levels [54].

## 5. The Missing Gap in the Knowledge

As mentioned above, SGLT2 inhibitors have several pleiotropic effects on inflammation, immune regulation, oxidative stress, and podocyte integrity. Many of the pathways affected have a possible pathogenetic role in the development and progression of primary or secondary glomerulonephritis, which theoretically suggests a specific effect. However, experimental evidence of this relationship is still limited and may be an area of exciting future research. Obtaining more specific data may also help to identify individual glomerulonephritis types that may benefit from SGLT2 inhibition.

DAPA-CKD [30] and EMPA-KIDNEY [28] have studied a large number of patients with glomerulonephritis. They provide solid evidence of efficacy and safety. However, due to the inclusion and exclusion criteria of the two trials, several questions remain regarding this specific patient population. Indeed, much of the evidence derives from post hoc analyses of trials that do not exclusively focus on glomerulonephritis. Also, data on lupus nephritis and ANCA vasculitis are sparse, relying on small cohorts or observational studies.

Furthermore, most patients enrolled in DAPA-CKD and EMPA-KIDNEY had CKD stage III or IV, with a small percentage having stage II and none with stage I. The choice of this patient population was made compulsory on a regulatory basis. Indeed, patients with established CKD are at higher risk of progressing towards ESKF or of experiencing a declining eGFR. Thus, they are optimal candidates for demonstrating treatment efficacy in a reasonable period. However, from a theoretical point of view, the excluded patients (i.e., those still having normal kidney function) are the ones more likely to experience a long-term benefit, as sclerosis and scarring have not developed yet. To demonstrate the efficacy of SGLT2 inhibitors in the early phases of kidney damage, studies on histological modifications or with specific biomarkers beyond proteinuria, such as CD80 or urinary TGF-β, could help with gaining further insights. Furthermore, the two trials excluded patients with severe nephrotic syndrome. It is thus unknown whether SGLT2 inhibitors maintain their efficacy in heavily proteinuric patients.

In everyday clinical practice, the antiproteinuric effect shown by DAP-CKD and EMPA-KIDNEY make SGLT2 inhibitors appealing for patients with glomerulonephritis. This is particularly true for patients with mild proteinuria. In this setting, a 30–40% decrease in proteinuria may translate into the transition of the patient from being at risk of progression to “clinical stability” without the need for immunosuppression. However, this “clinical stability” could be misleading, since this does not necessarily imply the immunological remission of the glomerulonephritis.

Currently, we have no direct comparison with other “supportive” therapies for glomerulonephritis. In particular, head-to-head studies comparing the effects of SGLT2 inhibitors with sparsentan or finerenone are missing. These trials will possibly never be designed because SGLT2 inhibitors are considered the gold standard. In this respect, it should be underlined that in IgAN patients, the antiproteinuric effect of sparsentan looks more potent than that observed with SGLT2 inhibitors [55]. Studies testing SGLT2 inhibitors combined with sparsentan or anti-aldosterone agents are underway.

SGLT2 inhibitors have been tested primarily in combination with RAS inhibitors; no specific information has been published on the subgroup of patients not treated with RAS inhibitors in the EMPA-KIDNEY trial with glomerulonephritis. For this reason, evidence is lacking on whether SGLTS inhibitors are effective without the concomitant presence of RAS blockade.

Another area for future investigation is whether the efficacy and safety of SGLT2 inhibitors are homogeneous across the class or whether there are differences between individual molecules. Indeed, the gliflozins have different degrees of selectivity for the SGLT1 and SGLT2 receptors, with empagliflozin being the most selective on the SGLT2 receptor, followed by dapagliflozin and ertugliflozin [56]. Conversely, canagliflozin also has significant SGLT1 inhibitory activity [56]. Finally, sotagliflozin is considered a dual SGLT1/2 inhibitor [56]. Theoretically, inhibition of the SGLT1 receptor may be beneficial. Individuals with a genetically reduced SGLT1 transporter are protected from developing heart failure and have a lower risk of all-cause mortality than those without mutations [57]. In addition, the SGLT1 transporter is expressed not only in the gut, where it is involved in glucose reabsorption, but also in the heart, kidneys, and skeletal muscle [57]. Finally, the more the molecule also targets SGLT1, the more complete the inhibition of glucose reabsorption along the renal tubules [57]. Specific to glomerulonephritis, canagliflozin has only been tested in patients with type 2 diabetes. Therefore, its use is limited to patients with both diseases. The available evidence from clinical trials has thus only been obtained with dapagliflozin and empagliflozin. No direct comparison of the two molecules has been made, and indirect comparison is made difficult by the different inclusion and exclusion criteria of the two clinical trials. Taking all these considerations into account, it is currently unknown whether empagliflozin and dapagliflozin might have different efficacy or safety in patients with glomerulonephritis.

In the last decades, several proteinuric patients with glomerulonephritis have been treated with the dual RAS blockade. The use of this treatment strategy has progressively declined over time and is now discouraged by the 2024 KDIGO Guidelines for the management and treatment of CKD [29]. Nevertheless, in our clinical practice, we have often observed a more consistent antiproteinuric effect of dual RAS blockade than an SGLT2 inhibitor plus one single RAS inhibitor [53]. Indeed, it is a common clinical observation that many patients shifting from dual blockade to one single RAS blocker plus an SGLT2 inhibitor experience increased proteinuria after months or years of stability at lower levels.

It is still controversial whether SGLT2 inhibitors are safe during active immunosuppression. This subset of patients was excluded from DAPA-CKD and EMPA-KIDNEY. Accordingly, in some countries, regulatory agencies do not reimburse SGLT2 inhibitors to patients receiving steroids or immunosuppressants. As shown in the previous section, data from small studies showed a relatively low rate of infections of the urinary tract. However, the choice of the patient is probably the key to ensuring safety. Older patients with many comorbidities, those receiving long-term immunosuppression, or those having advanced CKD are at higher risk for adverse events. More information will become available from ongoing trials targeting the complement system or lymphocyte activity in IgAN, where SGLT2 inhibitor use is permitted.

SGLT2 inhibitors influence the tubular transportation of calcium, magnesium, and phosphate. Even though meta-analyses have not confirmed that SGLT2 inhibitors may increase the risk of fracture, data are still needed in this respect in patients who have received or are in active treatment with steroids.

It is worth noting that even though the cardioprotective efficacy of SGLT2 inhibitors is well established and also confirmed in patients with glomerulonephritis, this subset of patients has a low burden of cardiovascular disease. Indeed, they are generally relatively young and, in most cases, without diabetes or heart failure. For this reason, the strength of the evidence on this point remains limited.

Finally, the findings from DAPA-CKD and EMPA-KIDNEY need to be confirmed in dedicated glomerulonephritis trials, including categories not included in DAPA-CKD and EMPA-KIDNEY (for example, those with normal renal function and nephrotic syndrome or those being treated with active immunosuppression). Trials are also needed in understudied populations with renal disease that may benefit particularly from SGLT2 inhibitors. The use of specific biomarkers of glomerular damage, inflammation, fibrosis, or immunological activity could help to better clarify whether SGLT2 inhibitors are “simply” nephroprotective agents or have beneficial effects specific to glomerulonephritis or, more broadly, to autoimmune disease. Glomerulonephritis is a class of disease and not a single entity; every single one has different pathogenetic mechanisms, onsets, clinical and histological manifestations, treatments, and outcomes. Experimental evidence specific to glomerulonephritis is thus needed to assess the direct effect of SGLT2 inhibitors in single animal models of glomerulonephritis. This would help to better deepen the direct effects on single aspects more specific than proteinuria reduction (podocyte integrity? mesangial inflammation? immunological activity? autoantibody production? complement activation?).

Despite the theoretical scientific interest, the ClinicalTrial.Gov website shows little activity around ongoing clinical trials in this specific patient population. Nevertheless, SGLT2 inhibitors are now considered the standard of care in ongoing trials of nephroprotective drugs. One example is SPARTUCS (NCT05856760), a 28-week, open-label, single-group exploratory study to determine the safety and efficacy of sparsentan in participants with IgAN despite having been on stable RAS and SGLT2 inhibitor treatment for at least 12 weeks prior to study entry. In addition, because of its potassium lowering effects, empagliflozin is currently being studied in combination with a new aldosterone synthase inhibitor, vicadrostat (BI 690517), or alone in EASY-KIDNEY (NCT06531824). This is a large, global, randomised Phase 3 clinical trial in CKD patients at risk of progression with or without type 2 diabetes. A significant proportion of the enrolled population is expected to have glomerulonephritis.

## 6. Conclusions

After over twenty years, SGLT2 inhibitors have joined RAS inhibitors as nephroprotective agents. Their combination has become the standard of care for many patients with CKD, including those with glomerulonephritis [29]. Apart from their effects driven by reduced sodium reabsorption, this new class of drugs may be particularly beneficial in patients with primary and secondary glomerulonephritis. They may not only slow down CKD progression and reduce proteinuria and cardiovascular risk, but they may also decrease the activity of glomerulonephritis because of their anti-inflammatory, antifibrotic, and immunomodulating activities. However, these pleiotropic effects are still speculative and thus not supported by clear, direct experimental evidence. Moreover, available clinical evidence for glomerulonephritis is mainly driven by secondary analyses excluding important categories of patients, namely those with normal renal function or proteinuria in the nephrotic range and those receiving active immunosuppression. In the latter subgroup, caution is needed, especially for those at high risk of infective complications of the urinary tract. At present, it is unknown whether SGLT2 inhibitors are superior to other nephroprotective agents, such as finerenone (now under clinical investigation for non-diabetic CKD) [58] and sparsentan for IgAN [59]. This question is only speculative, as there is a strong likelihood that SGLT2 inhibitors will be used in combination with them in the future.

## Figures and Tables

**Table 1:** The baseline characteristics of the subjects with glomerulonephritis enrolled in DAPA-CKD [30,31], EMPA-KIDNEY [28,32], and in the observational study from the Immunonephrology Working Group of the European Renal Association [33].

	DAPA-CKD N = 695	EMPA-KIDNEY N = 1669	IWG ^#^ N = 493
Age ^a^, years	~51.8 (13.8)	53·5 (13.6)	55 (42–65)
Sex female, N (%)	254 (36.5)	596 (35.7)	157 (32)
Race, N (%)			
White Black Asian Other	341 (49) 9 (1.2) 326 (47) 19 (2.7)	765 (45.8) 2.2 (1.3) 863 (51.7) 19 (1.1)	Mainly NA NA NA
BMI ^a^, kg/m^2^	NA	27.2 (5.8)	29 (26–33)
Kidney biopsy, N (%)		1312 (78.6)	493 (100)
RAS inhibitors, N (%)	691 (99)	1535 (92)	493 (100)
Diabetes, N (%)	97 (13.9)	172 (10.3)	147 (30)
Hypertension, N (%)	NA	NA	357 (72)
Previous history of CVD, N (%)	24 (3.4) *	144 (8.6)	81 (16)
Maintenance immunosuppression, N (%)	0 (0) ^c^	139 (8.3)	79 (16)
eGFR ^a^, ml/min/1.73 m^2^	~42.9 (11.9)	42.4 (17.8)	56 (39–82)
UACR ^b^, mg/g	~978 (540–1750)	700 (306–1428)	1287 (729–2294)

*: Heart failure, ^#^: Immunonephrology Working Group of the European Renal Association, ^a^: mean ± standard deviation, ^b^: median (interquartile range), ^c^: excluded from the trial, BMI: body mass index, RAS: renin–angiotensin system, eGFR: estimated glomerular filtration rate, UACR: urinary albumin creatinine ratio, NA: not available

**Table 2:** Absolute risk reduction and number needed to treat for primary outcomes in patients with glomerulonephritis enrolled in EMPA-KIDNEY [34] and DAPA-CKD [31].

Outcome	Events		Absolute Risk Reduction (%; 95% CI)	NNT (n; 95% CI)
	SGLT2i	Placebo		
EMPA-KIDNEY				
Primary composite outcome	117/853 (13.72%)	142/816 (17.40%)	3.69; 0.21–7.16	28; 14–481.8
Any kidney disease progression	115/853 (13.48%)	139/816 (17.30%)	3.55; 0.10–7.00	29; 14.3–980.8
ESKF	48/853 (5.6%)	58/816 (7.10%)	1.48; −0.86–3.83	NS
Sustained ≥40% eGFR decline	107/853 (12.54%)	136/816 (16.67%)	4.12; 0.73–7.51	25; 13.3–136.1
DAPA-CKD				
Primary composite outcome	22/343 (6.41%)	49/352 (13.92%)	7.51; 3.06–11.96	14; 8.4–32.7
Kidney specific composite outcome	21/343 (6.12%)	46/352 (13.07%)	6.95; 2.61–11.29	15; 8.9–38.4

SGLT2i, SGLT2 inhibitors; eGFR, estimated glomerular filtration rate; CKD, chronic kidney disease; NNT, number needed to treat; NS, non-significant; CI, confidence interval.
